# Clinical Relevance of Circulating Tumor Cells in Prostate Cancer Management

**DOI:** 10.3390/biomedicines9091179

**Published:** 2021-09-08

**Authors:** Wojciech A. Cieślikowski, Andrzej Antczak, Michał Nowicki, Maciej Zabel, Joanna Budna-Tukan

**Affiliations:** 1 Department of Urology, Poznan University of Medical Sciences, 61-285 Poznan, Poland; aa26@poczta.onet.pl; 2 Department of Histology and Embryology, Poznan University of Medical Sciences, 60-781 Poznan, Poland; mnowicki@ump.edu.pl (M.N.); jbudna@ump.edu.pl (J.B.-T.); 3 Department of Anatomy and Histology, Collegium Medicum, University of Zielona Gora, 65-046 Zielona Gora, Poland; mazab@ump.edu.pl; 4 Division of Histology and Embryology, Department of Human Morphology and Embryology, Wroclaw Medical University, 50-368 Wroclaw, Poland

**Keywords:** CTC, liquid biopsy, cancer biomarkers, prognostic value, predictive value, cancer, molecular characterization

## Abstract

Given the low specificity of the routinely used biomarker prostate-specific antigen, circulating tumor cell (CTC) enumeration seems to be particularly useful in the monitoring of prostate cancer. In this review, we focused on a few aspects of CTC enumeration in prostate malignancies: prognostic value in metastatic and non-metastatic tumors, role in the monitoring of treatment outcomes, use as a surrogate marker for survival, and other applications, mostly for research purposes. CTC enumeration, without a doubt, offers an attractive perspective in the management of prostate cancer. However, the vast majority of available data about the role of CTC in this malignancy originate from randomized studies of anticancer agents and do not necessarily translate into real-world clinical practice. Further, most studies on the application of CTC in prostate cancer patients were limited to advanced stages of this malignancy. Meanwhile, the role of CTC in the early stages of prostate cancer, in which some patients may present with occult disseminated disease, is still relatively poorly understood, and should thus be studied extensively. Other obstacles in the widespread application of CTC enumeration in routine clinical practice include considerable discrepancies in the number of cells determined with various commercially available systems.

## 1. Introduction

Prostate cancer is the most common male malignancy, with the global number of new cases being approximately 1.6 million annually, including ca. 400,000 new cases from Europe [1,2] and ca. 180,000 cases from the United States [3,4]. Risk factors of prostate cancer include genetic predisposition, family history of prostate and/or breast malignancies, and older age, with the median age at diagnosis of 72 years [5]. Approximately 79% of all prostate malignancies are diagnosed at local stages. In 12% of the patients, the diagnosis is established after regional lymph node involvement, and in another 5% after the development of local metastases [6]. Prognosis in prostate cancer depends primarily on the clinical stage. In patients with local or locoregional tumors, the likelihood of five-year survival is approximately 100%. However, the development of distant metastases is associated with a substantial decrease in five-year survival rate, down to about 30% [6]. Due to the relatively high incidence of prostate cancer, despite a quite favorable prognosis, this malignancy causes nearly 370,000 deaths annually [3], which makes it the fifth leading cause of cancer death worldwide [3].

## 2. Current Diagnostic and Monitoring Options

Prostate cancer is a highly genetically heterogeneous malignancy, with the spectrum of cases varying from indolent tumors that grow slowly or do not grow at all to highly progressive and aggressive castration-resistant prostate cancer (CRPC) [7]. According to current standards, the diagnosis of prostate cancer is established based on histopathological examination of the biopsy specimen, and prognosis and treatment depend on the biopsy Gleason score and serum concentration of prostate-specific antigen (PSA) [8]. Current diagnostic guidelines for biopsy recommend at least eight cores for glandular volumes of 30–40 mL [9], and with the transrectal approach 10–12 cores are most commonly used in clinical practice. The transperineal biopsy can be considered an alternative for patients that yielded a negative result from a transrectal biopsy but for whom prostate cancer cannot yet be excluded [10]. Magnetic resonance imaging (MRI) of the prostate is increasingly recognized as a noninvasive technique capable of detecting abnormal lesions that might be missed by transrectal ultrasound, especially in the anterior lobe [11]. Several biomarkers have been investigated to improve the detection of potentially aggressive prostatic tumors. One of the two available tests for prostate biopsy samples is Oncotype DX (Genomic Health Inc., Redwood City, CA, USA) which measures the expression of 12 cancer-related genes involved in four different biological pathways and five reference genes [12]. The result, expressed as a Genomic Prostate Score, is used to stratify the patients into very low-, low-, and intermediate-risk groups. Another molecular test is Polaris (Myriad Genetics Inc., Salt Lake City, UT, USA), which stratifies the risk of prostate cancer progression based on the expression of 46 different genes [13].

PSA is a protease secreted by prostate cancer cells. Although the serum concentration of this enzyme has been used for decades in diagnosing prostate cancer and monitoring treatment outcomes, this parameter has a serious limitation, namely low specificity. Elevated levels of PSA are also reported in many non-malignant diseases of the prostate, including benign hyperplasia, infections, and prostatic infarction [14]. The widespread use of serum PSA in detecting prostate cancer, beginning in the 1990s, contributed to a substantial shift in the distribution of clinical stages at the diagnosis, with ca. 95% of the malignancies diagnosed at local stages [15]. However, according to some researchers, this phenomenon might not reflect a beneficial trend towards earlier diagnosis of prostate cancer but might be instead associated with the overdiagnosis of this malignancy, with a high proportion of false-positive cases [16]. The latter notion is supported by the observation that in only 40–50% of patients with elevated serum PSA, the diagnosis of local prostate cancer is confirmed on the histopathological examination of biopsy specimens [6].

As many cases of prostate cancer detected based on elevated PSA concentration will not be necessarily aggressive, there is a need to identify a subset of patients with indolent tumors for whom active surveillance is a primary option until signs of disease progression are present. According to current clinical definitions, indolent prostate cancer is considered a low-risk (Gleason score ≤ 6, cT1c-cT2a, PSA ≤ 10 ng/mL) or very low-risk disease (Gleason ≤ 6, cT1c, PSA density ≤ 0.15 ng/mL/mg, involvement of ≤2 cores with ≤50% of each core, including the non-neoplastic intermediate segments) [17].

Aside from the diagnosis of prostate cancer, the serum concentration of PSA is also routinely determined in the monitoring of treatment outcomes. However, it needs to be stressed that PSA is encoded by an androgen-dependent gene. Thus, a change in serum androgen level or direct modulation of the PSA gene by some therapeutic agents used in the treatment of prostate cancer might contribute to a false decrease in PSA concentration, not associated with the cytoreduction [18].

## 3. Liquid Biopsy: Detection of Cell-Free DNA and Circulating Tumor Cells

Liquid biopsy (LB) is the real-time analysis of tumor cells or tumor cell products, such as cell-free circulating nucleic acids (cfDNA, cfRNA), extracellular vesicles, or proteins, released by primary or metastatic tumors into the blood or other body fluids [19]. LB opens new perspectives for the early detection of occult and recurrent malignancies, monitoring of treatment outcomes, and development of targeted therapies [20,21].

Cell-free DNA (cfDNA) is mostly released to the peripheral blood through necrosis and apoptosis [22]. It usually consists of 166 base pairs, which corresponds to the length of a DNA fragment wrapped around a nucleosome. While only a small portion of cfDNA (usually 0.01–5%) is released into the blood [20], ultrasensitive targeted techniques, such as droplet digital polymerase-chain reaction (ddPCR), BEAMing, and real-time polymerase-chain reaction (RT-PCR), are suitable for rapid and sensitive detection of prespecified cancer-associated mutations. Targeted next-generation sequencing methods, such as TAm-Seq, Safe-SeqS, and CAPP-Seq, can detect multiple rare mutations in cfDNA simultaneously [19]. In one study, cfDNA from patients with metastatic prostate cancer was shown to exhibit all driver DNA mutations present in matched metastatic tissue, along with some exclusive mutations [23]. However, mutation analysis has limited value in prostate cancer, given genomic heterogeneity and the frequent occurrence of structural gene arrangements in this malignancy. In contrast, several studies [24,25,26,27,28] demonstrated that early prostate cancer can be accurately detected based on the methylation of several genes in cfDNA from the blood or urine, including hypermethylation of *ST6GALNAC3*, *CCDC181,* and *HAPLN3* and promoter methylation of *APCme*, *FOXA1me*, *GSTP1me*, *HOXD3me*, *RARβ2me*, *RASSF1Ame*, *SEPT9me*, and *SOX17me*. cfDNA is also a valuable target for genomic aberrations of the androgen receptor gene, including mutations and amplifications or splice variants that can convey resistance to androgen deprivation therapy, and hence can be used to identify patients that might benefit more from other therapeutic approaches [29,30,31]. Unfortunately, the determination of cfDNA has also some drawbacks. Although targeted approaches are highly sensitive, they can detect mutations solely in a set of predefined genes. Meanwhile, untargeted approaches, such as whole genome sequencing or whole-exome sequencing, have lower sensitivity, require larger sample volumes, and are costly [32,33,34].

Circulating tumor cells (CTC) are the cells that detached from primary tumor mass or secondary metastatic tumor and reached peripheral circulation. As CTC are vital for tumor spread, they are considered a highly attractive prognostic and predictive biomarker and a measure of treatment outcomes [35]. The detection of CTC in peripheral blood, referred to as a “liquid biopsy”, constitutes a less invasive alternative to conventional tissue biopsy [36].

Available methods for CTC isolation and enumeration can be divided into two groups, epithelial cellular adhesion molecule (EpCAM)-dependent and EpCAM-independent [37,38,39]. The idea behind the EpCAM-dependent method is to capture and enrich CTC from peripheral blood through leukocyte depletion. In EpCAM-dependent systems, CTC are captured by magnetic beads coated with anti-EpCAM antibodies. One of the most widespread EpCAM-dependent systems is CellSearch^®^ (Menarini Silicon Biosystems). This is the only method approved by the US Food and Drug Administration (FDA) to monitor breast, colorectal, and prostate malignancies [40]. In the CellSearch^®^ system, CTC are defined as nucleated cells with 4′,6-diamidino-2-phenylindole (DAPI)-positive nucleus surrounded by cytokeratin-positive (CK+) and common leukocyte antigen-negative (CD45-) cytoplasm [40]. A study with the CellSearch^®^ confirmed that the cells isolated from the system’s cartridge, which satisfied the criteria mentioned above, shared their biological characteristics (expression of cytokeratin and alpha-methyl coenzyme A racemase, high frequency of aneusomy for chromosomes 17 and X, amplification of the androgen receptor gene) with prostate cancer cells [41]. The number of CTC determined with CellSearch^®^ is always expressed per a maximum of 7.5 mL of peripheral blood [40]. Due to such a small volume of blood being sampled, the sensitivity of the method is relatively lower [42]. This limitation of CellSearch^®^ has been partially neutralized in another EpCAM-dependent system, CellCollector (GILUPI). This is the first method to enumerate CTC in vivo, and hence, in a larger volume of blood. Captured CTC are identified and enumerated based on the intensity of cytokeratin immunofluorescence [43]. The most commonly used EpCAM-independent CTC enumeration system is EPithelial Immuno SPOT (EPISPOT). Unlike the two systems described above, EPISPOT allows differentiating between apoptotic and viable CTC at a single-cell level. CTC are identified based on the profile of proteins they release/express [44]. In the case of prostate cancer, the target proteins for EPISPOT are PSA and stem cell growth factor, FGF-2 [45].

Aside from the three commercially available systems mentioned above, CTC have been also retrieved with other methods, including reverse transcription-polymerase chain reaction targeting PSA, prostate-specific membrane antigen (PSM), prostate stem cell antigen (PSCA) and antioxidant genes, immunostaining for CK, CD45, androgen receptors and PSA, immune magnetic filtration, microfluidic methods, staining with near infrared dyes followed by fluorescence-activated cell sorting (FACS), detection of androgen receptor splice variant AR-V7 in serum samples, whole transcriptome amplification, multiplex ddPCR, and density gradient with immunostaining for PSA [6]. These methods constituted the basis for some novel isolation systems, the technical details of which have been reviewed recently by Casanova-Salas et al. [39].

Considering heterogeneous presentation, a considerable proportion of prostate malignancies diagnosed at local stages albeit without a possibility of excluding systemic spread, and the limitations of PSA mentioned above, CTC enumeration seems to be particularly useful in the monitoring of prostate cancer [46]. In this review, we focused on a few aspects of CTC enumeration in prostate malignancies: prognostic value in metastatic and non-metastatic tumors, role in the monitoring of treatment outcomes, use as a surrogate marker for survival, and other applications, mostly for research purposes.

## 4. Prognostic Value of CTC in Metastatic Prostate Cancer

Theoretically, the detection of CTC in peripheral blood of a prostate cancer patient would imply that prognosis is worse than in a CTC-negative person, also after adjustment for potential confounders, such as age, performance status, serum PSA, and others.

As mentioned above, one serious limitation of serum PSA as a biomarker for prostate cancer monitoring is the fact that this parameter is androgen dependent [18]. Meanwhile, CTC constitute a reliable marker of tumor spread, independent of androgen receptor-mediated signaling pathways [39].

In the case of the CellSearch^®^ system, the prognostic value of CTC is typically determined based on the cut-off limit specified in the US FDA registration document, i.e., ≥5 CTC per 7.5 mL of peripheral blood. This value is widely considered as an unfavorable prognostic factor in prostate cancer [18]. However, published evidence suggests that patients at various stages of prostate cancer treatment may differ considerably in terms of the number of CTC in their peripheral blood [47,48]. According to the literature, prior to first-line treatment, up to 75% of patients present with at least one CTC, but in only 25%, the CTC number is ≥5 cells per 7.5 mL [47,48]. Importantly, in one study, CTC number was shown to be a significant predictor of survival when analyzed as a continuous variable, especially together with serum PSA and albumin concentration [49]. In that study, involving 120 patients with metastatic prostate cancer, ≥5 CTC per 7.5 mL were detected in 57% of the cases. Higher CTC numbers were found in patients with bone metastases than in those with a soft tissue spread, as well as in persons with a history of prior cytotoxic chemotherapy. CTC numbers showed moderate correlations with the measures of the disease severity, such as serum PSA and bone scan index. However, the same study also demonstrated that the lack of CTC did not necessarily exclude a poor prognosis [49].

To summarize, published evidence suggests that the CTC number might satisfy the criteria of a prognostic marker in metastatic prostate cancer. However, one should consider that a considerable proportion of patients may not present with ≥5 CTC per 7.5 mL of peripheral blood, the cut-off value used traditionally to distinguish between more and less favorable prognosis. Thus, a reasonable direction of future research would be to identify a lower threshold value, more suitable for the purposes of establishing prognosis. Another issue that needs to be addressed is why some patients with metastatic prostate cancer have a worse prognosis despite the lack of detectable CTC in their peripheral blood. In other words, one should empirically verify the sensitivity and specificity of CTC number as a prognostic factor in metastatic prostate cancer.

## 5. CTC Number as a Marker of Treatment Response

The role of circulating tumor cells as a treatment response marker was first confirmed in the IMMC trial, in which CTC number turned out to be a better response marker than a 50% change in serum PSA already 2–5 weeks post-treatment [50]. Further analysis of the same data demonstrated that the prognostic value of CTC number considered as a continuous variable could be additionally improved when analyzed together with the baseline activity of lactated dehydrogenase (LDH), an established prognostic factor secreted by virtually all cancer cells [51]. Considering both these variables in a single model provided a better prognostic value than each of the single parameters.

Based on the abovementioned data and the fact that the CTC number is independent of the activity of androgen receptors, this parameter was considered as a response marker in trials of novel agents, abiraterone acetate (phase1 and 2 trials) [52,53,54], and enzalutamide [55]. All these studies confirmed the prognostic value of both detecting ≥5 CTC per 7.5 mL and conversion from ≥5 CTC to <5 CTC after treatment. Based on these findings, it has been suggested that the CTC number might be a surrogate marker of survival in phase 3 registration trials. Phase 3 trials also verified whether CTC number is a more accurate survival marker than the conventional prognostic model consisting of albumin, LDH, hemoglobin and alkaline phosphatase. Phase 2 and 3 trials of abiraterone acetate (COU-AA-301) and orteronel (ELM-PC4) demonstrated that the inclusion of CTC number improved the discrimination of patients with lower mortality risk but not those with higher risk, and resulted in better calibration, i.e., higher consistency of survival times prognosed based on the model with factual survival times [56].

These findings were then confirmed in multiple phase 2 trials and large phase 3 trials [57,58,59,60,61]. In post-treatment patients with metastatic CRPC (mCRPC), detection of ≥5 CTC per 7.5 mL of blood with CellSearch^®^ system was associated with inferior prognosis [59,60,61,62,63]. Although the studies mentioned above included a plethora of various treatment schemes, their results were surprisingly consistent, showing that the detection of ≥5 CTC per 7.5 mL was associated with shortening of survival time by approximately 50%, from 25 to 10.5 months [59], from 27.2 to 14 months [60], and from 26 to 13 months [61].

## 6. CTC Number as a Surrogate Survival Endpoint in Metastatic Prostate Cancer

The lack of early biomarkers for clinically important endpoints, such as survival, constitutes an obstacle in research on anticancer drugs for prostate malignancies [18]. The first large phase 3 trial analyzing CTC number as a potential surrogate marker of overall survival was the previously mentioned COU-AA-301 study, a prospective randomized phase 3 trial comparing abiraterone plus prednisone to placebo plus prednisone in post-chemotherapy patients with mCRPC [64]. The primary endpoint in this study was overall survival. Additionally, the study verified whether CTC number, analyzed as a sole parameter or in conjunction with LDH activity, could serve as a surrogate marker of overall survival. Out of 1195 patients participating in the trial, information about the baseline number of CTC and the CTC number after 12 weeks was available in 711 [65]. The panel of two biomarkers, CTC number and LDH activity, was shown to satisfy all four Prentice criteria as a surrogate marker of survival at a single patient level.

The accuracy of variously defined endpoints including CTC number and serum PSA as surrogate markers of overall survival was verified across five prospective randomized trials [41]. The cumulative analysis included the data of 6081 patients with mCRPC participating in COU-AA-301 [64], AFFIRM [66], ELM-PC-5 [67], ELM-PC-4 [68], and COMET-1 [69] studies. CTC number and serum PSA were determined at the baseline and after 13 weeks. The analysis included eight various endpoints: complete elimination of CTC after 12 weeks, conversion of CTC number from ≥5 to <5 cells after 12 weeks, and reduction of CTC number and serum PSA by 30%, 50%, or 70% each. The highest discrimination value was obtained in the case of the first two endpoints [70].

The results summarized above suggest that CTC number is a useful parameter to be included in clinical trials. However, as shown in the next section, these findings do not necessarily translate into routine clinical practice.

## 7. CTC Number in the Monitoring of Prostate Cancer Outcomes

It needs to be emphasized that the use of CTC number as a marker in clinical trials, including highly selected groups of patients, is completely different from the monitoring of the disease outcome in a real-life clinical setting. In the latter case, it is less important if the CTC number exceeds the arbitrarily defined cut-off point or not. Instead, physicians verify whether CTC are present in a given patient, if so in what number, and whether that number changes over the course of the disease [18]. Theoretically, one could expect that patients without CTC or with <5 CTC per 7.5 mL of blood have a better prognosis, and an increase in the CTC number to ≥5 over the course of the disease corresponds to progression/recurrence of prostate cancer. However, we do not have enough population data to ultimately conclude that the observations from clinical trials are entirely applicable to real-life practice [18]. Further, it needs to be stressed that, based on current evidence, the lack of conversion from ≥5 to <5 CTC per 7.5 mL or an increase in the CTC number do not justify a change of treatment regimen. Similarly, a decrease in CTC number below 5 per 7.5 mL should not be considered an ultimate proof of improvement in a real-life clinical setting [18]. Another issue that is yet to be addressed is whether a single increase/decrease in CTC number is clinically meaningful [71].

While the prognostic value of CTC number has been confirmed in the case of mCRPC, little is known about the role of this parameter in non-castration resistant metastatic prostate cancer (ncMPC). However, sparse published data suggest that, also in this type of prostate cancer, an increase in CTC number is associated with worse survival and faster progression [72,73,74]. In a prospective study including 30 patients with localized prostate cancer (LPC), 30 persons with ncMPC, and 30 healthy volunteers [72], the risk of mortality and progression for individuals with ≥4 CPC per 7.5 mL was 4.1 and 8.5 times higher, respectively. Patients with ≥4 CPC had significantly shorter overall survival and progression-free survival. Finally, CPC number ≥4 per 7.5 mL was an independent predictor of progression-free survival [72]. The same study, as well as another two, identified the presence of CTC in patients with ncMPC receiving androgen-deprivation therapy as an independent predictor of the magnitude and duration of treatment response, with the cut-off values for CTC number varying between ≥3 and 5 [72,73,74]. However, all studies mentioned above included small groups of patients with ncMPC, and hence their findings are yet to be verified.

## 8. CTC Number at Early Stages of Prostate Cancer

While relatively more is known about the role of CTC enumeration in mCRPC, available data on the significance of these cells at earlier stages of prostate cancer are sparse and inconclusive.

Research on the role of CTC in early LPC included patients with moderate to high risk of progression. Treatment responses in this group of patients vary considerably, and prognosis established based on currently available biomarkers, primarily serum PSA, is not necessarily accurate [18]. Studies verifying whether the inclusion of CTC number in the panel of biomarkers will be reflected by higher prognostic accuracy used the US FDA-approved CellSearch^®^ system. CTC number was determined prior to and after the treatment according to various schemes, including neoadjuvant chemotherapy with subsequent prostatectomy [75] and prostatectomy alone [76,77,78]. CTC number was also determined in 36 patients with LPC who showed the signs of biochemical recurrence after prostatectomy and/or radiotherapy [79]. Despite a considerable variance of the study groups in terms of patient characteristics and treatment regimens, the results were quite consistent. CTC were found rarely in LPC, and if present, their number was low (1–2 per 7.5 mL on average). The presence and number of CTC did not correlate with other clinicopathological parameters, such as Gleason score, serum PSA, and TNM stage [75,76,77,78,79]. The proportion of CTC-positive patients with LPC was lower than in CRPC, between 8% and 27%. However, most of the studies mentioned above included small numbers of patients and had an exploratory character. The largest study involved 152 patients with LPC qualified for radical prostatectomy [78]. At the baseline, CTC were detected in only 11% of patients (*n* = 17). No statistically significant relationship was found between the CTC number and the occurrence of biochemical recurrence during a 48-month follow-up period [78]. Moreover, a few studies showed that the frequency of CTC detection in patients with LPC and controls with serum PSA between 2.5 and 10 ng/mL was similar, approximately 20% [72,76,80].

The results summarized above does not seem to warrant a routine determination of CTC number in patients with LPC unless more sensitive diagnostic systems become available [81,82,83]. In this context, it is worth mentioning that published data about CTC numbers in LPC vary considerably and seem to depend on the type of enumeration system used. The authors of one study compared the number of CTC in peripheral blood of 107 patients with high-risk LPC prior to radical prostatectomy and three months after the procedure [81]. CTC were determined with all three commercially available systems, CellSearch^®^, CellCollector, and EPISPOT. The proportion of CTC-positive patients varied from 37% for CellSearch^®^ to 54.9% for CellCollector and 58.7% for EPISPOT. A significant post-prostatectomy decrease in the proportion of CTC-positive patients (66% vs. 34%) was demonstrated solely with the CellCollector system. The number of CTC determined with EPISPOT correlated significantly with serum PSA and the clinical stage of prostate cancer. Using all three systems, CTC were detected in 81.3% of the patients, including 21.5% patients with ≥5 CTC per 7.5 mL [81].

These findings are consistent with the results of our study involving a group of 104 patients with newly diagnosed high-risk prostate cancer, among them 19 with disseminated and 85 with localized disease [84]. The study also showed significant discrepancies in the number of CTC detected with various systems. Using the CellCollector, CTC were found in 57.7% of patients, with the aid of the EPISPOT in 52%, and by means of the CellSearch^®^ system in 23.9%. Patients with disseminated cancer presented with significantly higher CTC counts determined with the CellSearch^®^, but not with other systems. Although the vast majority (94.7%) of patients with disseminated prostate cancer tested positively for CTCs on at least one of the assays, an equally large proportion of the positive results was documented in the non-metastatic group [84].

Another study using a label-independent microfluidic platform for CTC isolation included 50 patients with LPC before radical prostatectomy [82]. CTC were detected in 50% of the patients, with the median number of nine cells (range: 1–417) per 2 mL of peripheral blood. However, similar to previously mentioned studies, no significant correlations were found between the CTC number and clinicopathological parameters [82].

Recently, in a study of 37 patients with LPC examined 2–5 months after radical prostatectomy, CTC were determined with label-independent Epic Sciences platforms [85]. CTC were detected in up to 81.1% of the patients, and their mean number was relatively high, 5.2 cells per ml of blood. The patients with higher CTC numbers tended to have shorter progression-free survival, which would imply that the Epic Sciences platform may be suitable for the detection of CTC as a prognostic marker in LPC [85].

## 9. Other Applications of CTC in Prostate Cancer

Recently, molecular markers of prostate cancer gain a growing interest of researchers. The most commonly observed genetic defects found in this malignancy are disturbances on the androgen receptor (AR) pathway [86,87,88]. Published evidence suggests that CTC mirror accurately AR gene amplification status in conventional biopsy specimens [89], and hence, could be used as a less invasive source of material for molecular research. In one study using such a material, a significant correlation was observed between the nuclear localization of the AR within the CTC and resistance to taxanes [90].

Recently, AR splice variants, especially ARV7, were shown to be associated with resistance to some hormonal agents, such as enzalutamide and abiraterone [91]. The AR-V7 protein is known to be rarely expressed in primary prostate cancer but is found in 75% of mCRPCs [92]. Importantly, a link has been shown between the detection of ARV7 mRNA in CTC, lower PSA response rates, shorter progression-free survival and overall survival in patients with mCRPC receiving enzalutamide or abiraterone [91]. Another study conducted by the same group demonstrated that CTC-negative patients had the most favorable outcome after the first line and second line hormone therapy, followed by CTC-positive/ARV7-negative patients and CTC-positive/ARV7-positive ones [93]. The results of the studies mentioned above, as well as the data provided by other authors [94,95] led to the first approval of CTC as a predictive biomarker to guide the choice of a taxane versus an androgen receptor signaling inhibitor (ARSi) in the second-line or greater setting. While the presence of ARV7 mRNA and ARV7 protein in CTC can be considered a blood-based biomarker to predict de novo or developed resistance to andro- gen pathway-targeted therapies [96], this method is not free from potential drawbacks. Importantly, the ARV7 status can change during the course of antiandrogen therapy [97,98,99], and the ARV7 status in individual CTCs from the same patient is heterogeneous [100]. This all might contribute to treatment resistance. Further, a growing body of evidence suggests that ARV7 status alone does not entirely explain the resistance observed in a clinical setting [101,102].

The results of one recent study suggest that aside from predicting treatment responses, genomic profile of CTC may be also associated with the aggressiveness of prostatic malignancies. Specifically, it has been shown that the loss of at least two tumor suppressor genes from at least one CTC was associated with poor survival and increased genomic instability [103]. These findings support single CTC sequencing as a non-invasive method to characterize cancer heterogeneity.

## 10. Conclusions

CTC enumeration, without a doubt, offers an attractive perspective in the management of prostate cancer. It needs to be stressed that the population of prostate cancer patients includes ca. 15–20% of subjects with high-risk malignancies associated with a very high (ca. 80%) likelihood of systemic spread, primarily to the bones [104]. Even more importantly, even up to 20% of prostate cancers may be disseminated already at the time of primary diagnosis [105]. Hence, unlike in other malignancies in which it will supposedly remain an accessory test, CTC enumeration has the potential to become a primary diagnostic modality in prostate cancer. However, as outlined above and summarized in Table 1, this method has both pros and cons. The vast majority of available data about the role of CTC in prostate cancer originate from randomized studies of anticancer agents and do not necessarily translate into real-world clinical practice. Further, most studies on the application of CTC in prostate cancer patients were limited to advanced stages of this malignancy, in which the evidence of tumor spread can be confirmed with another method. Meanwhile, the role of CTC in the early stages of prostate cancer, in which some patients may already present with occult disseminated disease, is still relatively poorly understood and should be studied extensively. Another obstacle in the widespread application of CTC enumeration in routine clinical practice are considerable discrepancies in the number of cells determined with various commercially available systems. Available evidence suggests that capturing an adequate number of cells for determining the prognostic or predictive value of CTC would require the simultaneous use of more than one system [81], which with no doubt is not cost-effective. Hence, a foundation of future research on the application of circulating tumor cells in prostate cancer should be the optimization of a method for CTC enumeration. While liquid biopsies definitely represent a great value as biomarkers in prostate cancer, the standardization of assays and clinical validation is necessary prior to their implementation into routine practice.

## Figures and Tables

**Table 1:** Summary of pros and cons for various applications of CTC enumeration in prostate cancer (PC) management.

Application	Pros	Cons
Prognostic factor in metastatic PC	Some evidence suggests that ≥5 CTC per 7.5 mL might be an unfavorable prognostic factor [18]	Only approximately 25% of patients with metastatic PC have ≥5 CTC per 7.5 mL [47,48] Some patients without CTC have a worse prognosis [49] Unclear whether the CTC number should be considered as a binary or continuous variable [49]
Marker of treatment response in metastatic PC	Multiple clinical trials demonstrated that the CTC number (≥5 vs. <5 per 7.5 mL) correlates with survival after treatment [57,58,59,60,61,62,63] Inclusion of the CTC number as a response marker improved the discrimination and calibration of survival models [56]	None
Surrogate survival endpoint in metastatic PC	CTC number (≥5 vs. <5 per 7.5 mL) was confirmed as an accurate surrogate survival endpoint in multiple clinical trials [64,65,66,67,68,69,70]	None
Marker in the monitoring of PC outcomes	In some small studies, a higher CTC number correlated with worse survival and faster progression [72,73,74]	Not enough data from large studies confirming prognostic value of the CTC number No data justifying a change of treatment regimen in patients with without the CTC response/increase in the CTC number The cut-off value of ≥5 CTC per 7.5 does not seem to be applicable to clinical setting [18] Unclear whether a single change in CTC number is clinically relevant [71]
Prognostic factor in localized PC	Theoretically suitable for the identification of patients with occult disseminated disease	CTC rarely found in patients with localized PC [75,76,77] CTC number does not seem to correlate with other clinicopathological parameters [75,76,77,78,79] Discrepancies in the CTC numbers obtained with various methods negatively affect cost-effectiveness of this parameter [81,82,83,84]
Source of the genetic material of PC	CTC mirror accurately genetic status of cancer cells found in biopsy specimens [89] Genetic status of CRC was shown to be a predictor of prostate cancer aggressiveness [103] and therapeutic responses [90,91,92,93,94,95,96,97,98,99,100,101,102]	Not enough evidence confirming superiority of this method over conventional biopsy

## Data Availability

Not applicable.
